# Assessment of Frailty and Association With Progression of Benign Prostatic Hyperplasia Symptoms and Serious Adverse Events Among Men Using Drug Therapy

**DOI:** 10.1001/jamanetworkopen.2021.34427

**Published:** 2021-11-24

**Authors:** Scott R. Bauer, Louise C. Walter, Kristine E. Ensrud, Anne M. Suskind, John C. Newman, William A. Ricke, Teresa T. Liu, Kevin T. McVary, Kenneth Covinsky

**Affiliations:** 1 Division of General Internal Medicine, Department of Medicine, University of California, San Francisco; 2 Department of Urology, University of California, San Francisco; 3 San Francisco Veterans Affairs Medical Center, San Francisco, California; 4 Division of Geriatrics, Department of Medicine, University of California, San Francisco; 5 Department of Medicine and Division of Epidemiology and Community Health, University of Minnesota, Minneapolis; 6 Center for Care Delivery and Outcomes Research, Veterans Affairs Health Care System, Minneapolis, Minnesota; 7 Buck Institute for Research on Aging, Novato, California; 8 George M. O’Brien Center of Research Excellence, Department of Urology, University of Wisconsin School of Medicine and Public Health, Madison; 9 Department of Urology and Center for Male Health, Stritch School of Medicine and Loyola University Medical Center, Maywood, Illinois

## Abstract

**Importance:**

Benign prostatic hyperplasia (BPH) in older men can cause lower urinary tract symptoms (LUTS), which are increasingly managed with medications. Frailty may contribute to both symptom progression and serious adverse events (SAEs), shifting the balance of benefits and harms of drug therapy.

**Objective:**

To assess the association between a deficit accumulation frailty index and clinical BPH progression or SAE.

**Design, Setting, and Participants:**

This cohort study used data from the Medical Therapy of Prostatic Symptoms trial, which compared placebo, doxazosin, finasteride, and combination therapy in men with moderate-to-severe LUTS, reduced urinary flow rate, and no prior BPH interventions, hypotension, or elevated prostate-specific antigen. Enrollment was from 1995 to 1998, and follow-up was through 2001. Data were assessed in February 2021.

**Exposures:**

A frailty index (score range, 0-1) using 68 potential deficits collected at baseline was used to categorized men as robust (score ≤0.1), prefrail (score 0.1 to <0.25), or frail (score ≥0.25).

**Main Outcomes and Measures:**

Primary outcomes were time to clinical BPH progression and time to SAE, as defined in the parent trial. Adjusted hazard ratios (AHRs) were estimated using Cox proportional hazards regressions adjusted for demographic variables, treatment group, measures of obstruction, and comorbidities.

**Results:**

Among 3047 men (mean [SD] age, 62.6 [7.3] years; range, 50-89 years) in this analysis, 745 (24%) were robust, 1824 (60%) were prefrail, and 478 (16%) were frail at baseline. Compared with robust men, frail men were older (age ≥75 years, 12 men [2%] vs 62 men [13%]), less likely to be White (646 men [87%] vs 344 men [72%]), less likely to be married (599 men [80%] vs 342 men [72%]), and less likely to have 16 years or more of education (471 men [63%] vs 150 men [31%]). During mean (SD) follow-up of 4.0 (1.5) years, the incidence rate of clinical BPH progression was 2.2 events per 100 person-years among robust men, 2.9 events per 100 person-years among prefrail men (AHR, 1.36; 95% CI, 1.02-1.83), and 4.0 events per 100 person-years among frail men (AHR, 1.82; 95% CI, 1.24-2.67; linear *P* = .005). Larger point estimates were seen among men who received doxazosin or combination therapy, although the test for interaction between frailty index and treatment group did not reach statistical significance (*P *for interaction = .06). Risk of SAE was higher among prefrail and frail men (prefrail vs robust AHR, 1.81; 95% CI, 1.48-2.23; frail vs robust AHR, 2.86; 95% CI, 2.21-3.69; linear *P* < .001); this association was similar across treatment groups (*P *for interaction = .76).

**Conclusions and Relevance:**

These findings suggest that frailty is independently associated with greater risk of both clinical BPH progression and SAEs. Older frail men with BPH considering initiation of drug therapy should be counseled regarding their higher risk of progression despite combination therapy and their likelihood of experiencing SAEs regardless of treatment choice.

## Key Points

**Question:**

Is frailty associated with increased risk of progression of benign prostatic hyperplasia (BPH) symptoms and serious adverse events among men receiving drug therapy?

**Findings:**

In a cohort study of 3047 men with moderate-to-severe lower urinary tract symptoms due to suspected BPH, higher deficit accumulation frailty index was associated with a higher risk of clinical BPH progression, particularly among men receiving doxazosin with finasteride, and serious adverse events.

**Meaning:**

These findings suggest that frail men with lower urinary tract symptoms due to suspected BPH are more likely to have progression of symptoms, despite drug therapy, as well as serious adverse events.

## Teaser

This cohort study examines the association between a deficit accumulation frailty index and clinical progression or serious adverse events among men using drug therapy for benign prostatic hyperplasia (BPH).

## Introduction

Bladder outlet obstruction due to benign prostatic hyperplasia (BPH), the histological process that leads to an enlarged prostate, is a common cause of male lower urinary tract symptoms (LUTS). Currently, one-third of men older than 75 years with newly diagnosed LUTS will receive BPH drug therapy, twice the rate of younger men.^1^

Combination therapy with α-adrenergic-receptor antagonist (α-blocker) plus a 5α-reductase inhibitor has been the standard drug therapy for BPH for over 2 decades since the landmark Medical Therapy of Prostatic Symptoms (MTOPS) trial demonstrated lower rates of clinical BPH progression, primarily symptom progression and acute urinary retention, compared with men receiving placebo.^2^ Drug therapy decreases the risk of clinical BPH progression by decreasing smooth-muscle tone in the prostate and bladder neck (via α-blockers) and decreasing prostate volume (via 5α-reductase inhibitors).^3^ However, LUTS among older men with and without BPH are extremely common and heterogenous, with multifactorial causes,^4,5^ contributing to widespread and long-term use of BPH medications.^6,7,8^ Medical BPH therapy is also associated with several important adverse drug events that are particularly harmful for older men, including orthostatic hypotension, falls, depression, dementia, and suicidal ideation.^9,10,11,12,13^ Still unknown is whether there are identifiable subsets of older men (eg, those that are frail) for whom the risks of long-term drug therapy for BPH outweigh benefits.

Frailty, as defined by the accumulation of health and functional problems (ie, deficit accumulation frailty), is associated with morbidity and mortality because of a greater vulnerability to stressors and is considered a marker of biological, rather than chronological, age.^14,15^ Although associations between chronological age and male LUTS or BPH are well established,^8,16,17,18^ including data from the MTOPs trial,^19^ frailty and other markers of biological age may represent more important risk factors than chronological age alone.^20,21,22,23,24,25^ However, prior studies are limited by cross-sectional design, lack of a clinical diagnosis or objective measures of benign prostatic obstruction and frailty, BPH interventions that were neither randomized nor placebo controlled, and no formal interaction testing.

This cohort study using data from the MTOPS trial evaluates the longitudinal association of a deficit accumulation frailty index with risk of clinical BPH progression and serious adverse events (SAEs), using standardized definitions for both. We hypothesized that greater frailty index assessed at baseline would be independently associated with higher rates of both clinical BPH progression and SAE and that both associations would be highest among men receiving combination therapy.

## Methods

### Participants

The MTOPS study design has been previously described.^2,26^ Participants provided written informed consent, and institutional review boards at each participating center approved the study. Our local institutional review board deemed this study to be not human participants research because we only had access to deidentified data. This analysis follows the Strengthening the Reporting of Observational Studies in Epidemiology (STROBE) reporting guidelines for observational studies.^27^

Men aged 50 years and older were recruited from December 1995 to March 1998 and were enrolled if they reported moderate-to-severe LUTS (American Urological Association Symptom Index [AUASI] score, 8-30), demonstrated a reduced urinary flow rate (maximum of 4-15 mL/s), and denied a history of surgical treatment or recent drug therapy for BPH, hypotension (defined as supine blood pressure below 90/70 mm Hg), or elevated prostate-specific antigen (>10 ng/mL [to convert to micrograms per liter, multiply by 1]). Men taking anticholinergic medications for any reason except glaucoma were also excluded. The eligible participants were randomized to receive placebo, doxazosin monotherapy, finasteride monotherapy, or combination therapy with both agents, and were followed for a mean (SD) of 4.0 (1.5) years. Follow-up time was calculated from date of randomization until the first event of clinical BPH progression, SAE, invasive therapy related to BPH (252 participants underwent transurethral prostatectomy, transurethral incision of the prostate, laser therapy, stenting, open prostatectomy, or transurethral microwave therapy), prostate cancer (167 participants), bladder cancer (12 participants), or death (127 participants).

### Clinical BPH Progression

Clinical BPH progression was defined as a composite end point including the occurrence of any of the following: LUTS progression (increase from baseline of at least 4 points in the AUASI), acute urinary retention (inability to urinate, requiring catheterization in the absence of an obvious cause other than BPH, such as anesthesia), urinary incontinence, recurrent urinary tract infection or urosepsis, or an increase in serum creatinine attributable to BPH of at least 1.5 mg/dL (to convert to micromoles per liter, multiply by 88.4) and to a value at least 50% above baseline.^2^ The AUASI and other composite end points were assessed every 3 months. All composite outcomes were adjudicated by clinical review committee.^2^

### Serious Adverse Events

SAEs were defined as fatal or life-threatening, permanently disabling, requiring or prolonging inpatient hospitalization, a congenital anomaly or cancer, an overdose, or any medical event that jeopardized the patient on the basis of appropriate medical judgment and may have required medical or surgical intervention to prevent an adverse outcome. Adverse events caused by doxazosin and/or finasteride, but also other causes occurring at a similar rate among participants randomized to placebo, were assessed every 3 months and adjudicated by a data and safety monitoring board.

### Frailty Index

We followed a standardized approach to creating a frailty index based on deficit accumulation using data collected at baseline.^28^ We started with deficits from the Systolic Blood Pressure Intervention Trial frailty index^29^ and added deficits from another frailty index based on abnormal laboratory values.^30^ Finally, we included additional self-reported conditions, functional status, and symptoms associated with increasing age, with multifactorial causes, and associated with negative health outcomes. The final frailty index included 68 items (eTable 1 in the Supplement), each weighted equally, and was calculated as the sum of the score for each item (range, 0-1) divided by total number of nonmissing items. All participants had more than 30 nonmissing items and were included in the primary analysis. The frailty index categorized participants as robust (score ≤0.1), prefrail (score 0.1 to <0.25), or frail (score ≥0.25).^31^ To confirm that the frailty index was associated with known constructs as expected among MTOPS participants, we determined that higher frailty index scores were associated with both older age and mortality (data not shown).

### Other Covariates

Demographic characteristics, including race and ethnicity, marital status, and education, were assessed at baseline. Race and ethnicity were self-identified by the participants and were assessed in this study because both deficit accumulation frailty and risk of BPH progression vary according to these demographic factors. Objective measures of benign prostatic obstruction, including maximum urinary flow rate (with a voided volume of ≥125 mL), serum prostate-specific antigen level, postvoid residual, and prostate volume (ellipsoid volume of total prostate gland assessed by transrectal ultrasonography^32^) were assessed at baseline by technicians trained and monitored by the MTOPS Biostatistical Coordinating Center.

### Statistical Analysis

For this post hoc analysis, the primary independent variable was deficit accumulation frailty index, and the 2 primary dependent variables were clinical BPH progression and SAE. LUTS progression and acute urinary retention, the most common composite events, were considered secondary outcomes. We first compared distributions of established LUTS and BPH risk factors across categories of frailty status using analysis of variance and 2-sided χ^2^ tests, as appropriate. We then generated cumulative incidence curves for each dependent variable stratified by frailty status and tested for differences between the curves using log-rank tests. To test the hypothesis that greater frailty index at baseline is independently associated with a greater risk of clinical BPH progression, we used adjusted Cox proportional hazards regression models because of the fixed follow-up period. We used the same approach to test the hypothesis that greater frailty index score at baseline is independently associated with SAE.

To adjust for confounders, we sequentially added groups of covariates to multivariable models. First, we adjusted for chronological age at enrollment. We further adjusted for treatment group plus objective markers of benign prostatic obstruction (prostate volume, postvoid residual, and maximum flow rate). In the fully adjusted model, we adjusted for age, treatment group, markers of benign prostatic obstruction, demographic variables (race and ethnicity, marital status, and education), body mass index (calculated as weight in kilograms divided by height in meters squared), and comorbidities (heart disease, hypertension, diabetes, pulmonary disease, neurological disease, and gastrointestinal disease). We assessed for modification of the main associations by including a cross-product term of frailty index (continuous) by treatment group (any drug vs placebo). We also conducted a sensitivity analysis excluding men with any missing frailty index items.

*P* < .05 was considered statistically significant in all analyses, which were performed using Stata statistical software version 15.1 (StataCorp). Data were assessed in February 2021.

## Results

Among 3047 men in the analytic cohort (mean [SD] age, 62.6 [7.3] years; range, 50-89 years), 745 (24%) were robust, 1824 (60%) were prefrail, and 478 (16%) were frail at baseline (Table 1). Compared with robust men, frail men were older (age ≥75 years, 12 men [2%] vs 62 men [13%]), less likely to be White (646 men [87%] vs 344 men [72%]), less likely to be married (599 men [80%] vs 342 men [72%]), and were less likely to have 16 years or more of education (471 men [63%] vs 150 men [31%]). In terms of BPH disease severity at baseline, frail participants had higher AUASI scores, including both voiding and storage subscores, higher maximum flow rate, and lower postvoid residuals compared with robust participants, but prostate volume and serum prostate-specific antigen level were similar across frailty categories (Table 1). Components of the frailty index also varied as expected by frailty status; frail men had higher body mass index and systolic blood pressure, worse renal function, worse physical and mental health, took more medications, and had a greater burden of comorbidities compared with robust men.

**Table 1.** Baseline Characteristics of Study Population, by Frailty Status

Characteristic	Participants, No. (%) (N = 3047)			*P* value^a^
	Robust (FI score ≤0.1)	Prefrail (FI score 0.1-0.25)	Frail (FI score ≥0.25)	
Sample size	745 (24)	1824 (60)	478 (16)	
Demographic variables				
Age, y				
50 to <55	153 (21)	277 (15)	47 (10)	<.001
55 to <60	184 (25)	374 (21)	87 (18)	
60 to <65	197 (26)	436 (24)	106 (22)	
65 to <70	131 (18)	379 (21)	108 (23)	
70 to <75	68 (9)	244 (13)	68 (14)	
≥75	12 (2)	114 (6)	62 (13)	
Race or ethnicity				
Black	46 (6)	164 (9)	60 (13)	<.001
Hispanic	41 (6)	115 (6)	67 (14)	
White	646 (87)	1519 (83)	344 (72)	
Other^b^	12 (2)	26 (1)	7 (2)	
Married	599 (80)	1396 (77)	342 (72)	.002
Education, y				
<12	29 (4)	139 (8)	91 (19)	<.001
12 to <16	245 (33)	806 (44)	237 (50)	
≥16	471 (63)	879 (48)	150 (31)	
Markers of health status, mean (SD)				
Body mass index^c^	26.4 (3)	27.9 (4)	29.1 (5)	<.001
Systolic blood pressure, mm Hg	128 (14)	136 (17)	141 (18)	<.001
Estimated glomerular filtration rate, mL/min/1.73 m^2^	79 (13)	77 (15)	76 (17)	<.001
MOS SF-36 Physical Component Score	56 (3)	51 (6)	38 (9)	<.001
MOS SF-36 Mental Component Score	56 (4)	53 (8)	46 (12)	<.001
Medications, No.				
Mean (SD)	1.3 (2)	2.1 (2)	3.1 (2)	<.001
Median (IQR)	1 (0-2)	2 (0-3)	3 (1-4)	
Self-reported comorbidities				
Heart disease	57 (8)	366 (20)	170 (36)	<.001
Hypertension	81 (11)	541 (30)	249 (52)	<.001
Diabetes	13 (2)	149 (8)	98 (21)	<.001
Pulmonary disease	29 (4)	220 (12)	87 (18)	<.001
Neurological disease	14 (2)	109 (6)	29 (6)	<.001
Gastrointestinal disease	92 (12)	559 (31)	155 (32)	<.001
Cancer	4 (1)	76 (4)	43 (9)	<.001
Benign prostatic hyperplasia disease severity, mean (SD)				
American Urological Association Symptom Index				
Total score	16.2 (6.0)	16.9 (6.0)	18.2 (6.0)	<.001
Voiding subscore	7.1 (3.0)	7.6 (3.0)	8.2 (3.0)	<.001
Storage subscore	9.1 (4.0)	9.3 (4.0)	10.0 (4.0)	<.001
Prostate volume, mL^d^	35 (20)	37 (20)	37 (21)	.15
Maximum urinary flow rate, mL/s	10.3 (3.0)	10.5 (3.0)	10.7 (3.0)	.02
Postvoid residual, mL				
Mean (SD)	65 (79)	72 (86)	60 (78)	.009
Median (IQR)	39 (12-86)	42 (13-100)	33 (11-81)	.01
Serum prostate-specific antigen, ng/mL	2.3 (2.0)	2.4 (2.0)	2.4 (2.0)	.61

Abbreviations: FI, frailty index; MOS SF-36, Medical Outcome Study Short Form (36-item).

SI conversion factor: To convert prostate-specific antigen to micrograms per liter, multiply by 1.

^a^ Calculated using analysis of variance or Kruskal-Wallis test for continuous variables and Pearson χ^2^ test for categorical or binary variables.

^b^ Other refers to Alaska Native, American Indian, Asian, or Pacific Islander.

^c^ Body mass index is calculated as weight in kilograms divided by height in meters squared.

^d^ Prostate volume was measured by transrectal ultrasonography.

**Figure 1.**
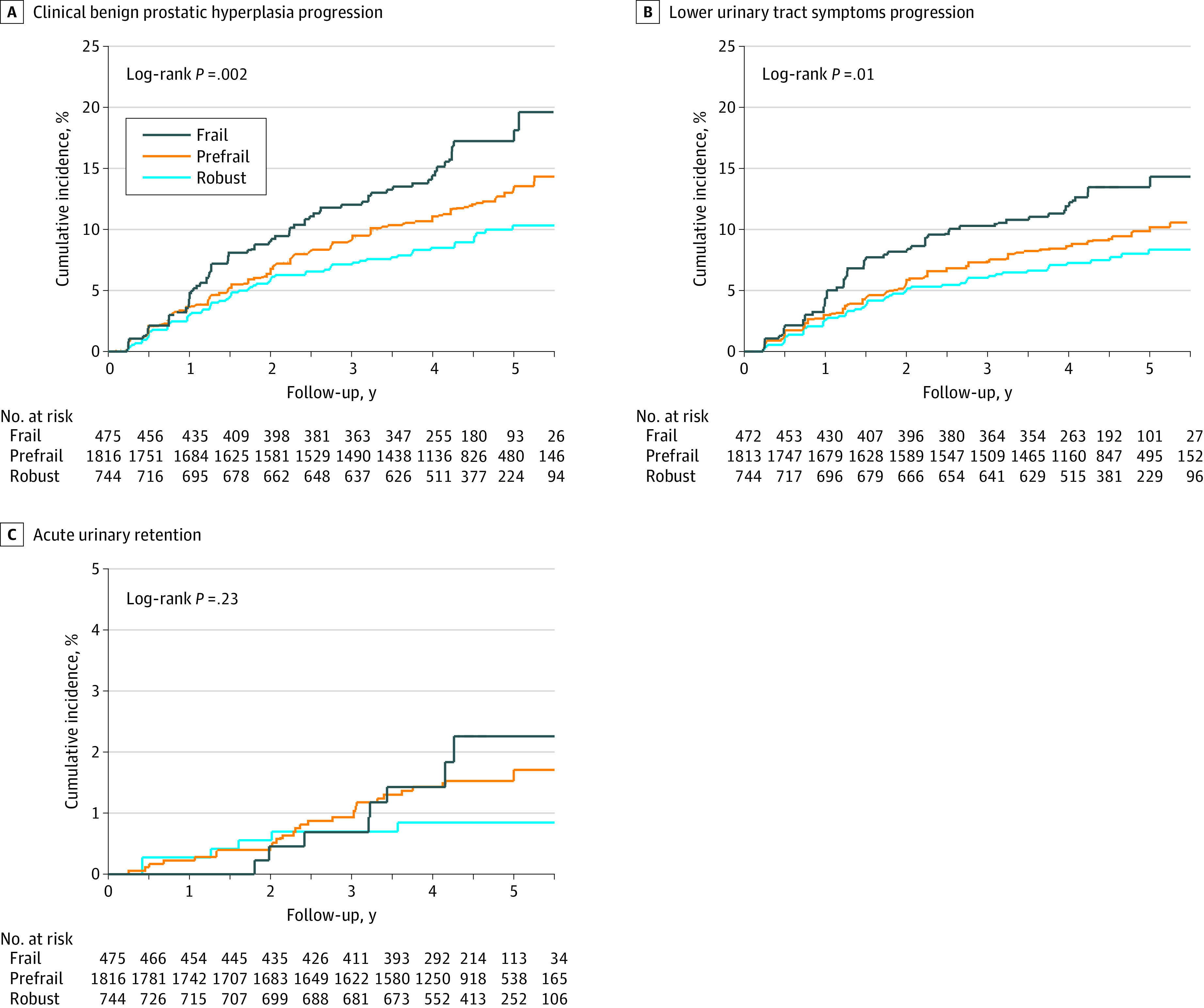
Cumulative Incidence Curves for Clinical Benign Prostatic Hyperplasia (BPH) Progression, Lower Urinary Tract Symptoms Progression, and Acute Urinary Retention, by Frailty Status Clinical BPH progression is defined according to the original trial as the occurrence of any of the following: lower urinary tract symptom progression (an increase from baseline of at least 4 points in the American Urological Association symptom index), acute urinary retention (the inability to urinate requiring catheterization in the absence of an obvious cause of acute retention other than BPH, such as anesthesia), urinary tract infection or urosepsis, incontinence, or an increase in the serum creatinine level, attributable to BPH, of at least 1.5 mg/dL (to convert to micromoles per liter, multiply by 88.4) and to a value at least 50% above baseline values.

Figure 1 displays cumulative incidence curves for clinical BPH progression (351 events), LUTS progression (274 events [78% of men]), and acute urinary retention (41 events [12% of men]), stratified by frailty status. In addition, 31 men (9%) developed urinary incontinence, less than 1% developed recurrent infections or urosepsis (5 men), and 0 had an increase in serum creatinine attributable to BPH. Table 2 reports the association of frailty with clinical BPH progression as well as separate associations with the 2 most common event types, LUTS progression and acute urinary retention. The incidence rates of clinical BPH progression were 2.2 events per 100 person-years (LUTS progression, 1.8 events per 100 person-years; acute urinary retention, 0.2 event per 100 person-years) among robust men, 2.9 events per 100 person-years (LUTS progression, 2.2 events per 100 person-years; acute urinary retention, 0.3 event per 100 person-years) among prefrail men, and 4.0 events per 100 person-years (LUTS progression, 3.2 events per 100 person-years; acute urinary retention, 0.4 event per 100 person-years) among frail men. Compared with robust men, the risk of clinical BPH progression was 36% and 82% higher among prefrail and frail men, respectively (prefrail vs robust adjusted hazard ratio [AHR], 1.36; 95% CI, 1.02-1.83; frail vs robust AHR, 1.82; 95% CI, 1.24-2.67; linear *P* = .005). Compared with robust men, the risk of LUTS progression was 29% and 79% higher among prefrail and frail men, respectively (prefrail vs robust AHR, 1.29; 95% CI, 0.93-1.78; frail vs robust AHR, 1.79; 95% CI, 1.16-2.75; linear *P* = .02). Among participants who experienced LUTS progression, the mean (SD) increase in AUASI voiding subscore from baseline was 4.9 (2.8) among robust men and 4.1 (2.5) among frail men, whereas the mean (SD) increase in AUASI storage subscore from baseline was 2.5 (2.3) among robust men and 2.9 (1.9) among frail men (eTable 2 in the Supplement). In sensitivity analyses restricted to men with 0 missing frailty index items, higher frailty index scores remained associated with greater risk of both clinical BPH and LUTS progression (eTable 3 in the Supplement). Frailty index was not significantly associated with acute urinary retention in any models, although acute urinary retention was a rare event in all treatment groups.

**Table 2.** Association of Frailty With Clinical BPH Progression, LUTS Progression, Acute Urinary Retention, and Serious Adverse Events in MTOPS

Varirable	Frailty status			Per 1 SD of FI	Linear *P* value
	Robust (FI score ≤0.1)	Prefrail (FI score 0.1-0.25)	Frail (FI score ≥0.25)		
Clinical BPH progression^a^					
Incidence rate, events/100 person-years (95% CI)	2.2 (1.7-2.7)	2.9 (2.5-3.3)	4.0 (3.2-5.1)	NA	NA
Unadjusted HR (95% CI)^b^	1 [Reference]	1.33 (1.01-1.74)	1.83 (1.31-2.55)	1.18 (1.07-1.30)	.001
Partially adjusted HR (95% CI)^c^	1 [Reference]	1.30 (0.99-1.72)	1.73 (1.23-2.43)	1.16 (1.05-1.29)	.004
Fully adjusted HR (95% CI)^d^	1 [Reference]	1.36 (1.02-1.83)	1.82 (1.24-2.67)	1.18 (1.05-1.33)	.005
LUTS progression					
Incidence rate, events/100 person-years (95% CI)	1.8 (1.4-2.3)	2.2 (1.9-2.6)	3.2 (2.4-4.1)	NA	NA
Unadjusted HR (95% CI)^b^	1 [Reference]	1.22 (0.90-1.65)	1.73 (1.20-2.51)	1.17 (1.05-1.30)	.006
Partially adjusted HR (95% CI)^c^	1 [Reference]	1.22 (0.89-1.66)	1.67 (1.14-2.44)	1.15 (1.03-1.29)	.01
Fully adjusted HR (95% CI)^d^	1 [Reference]	1.29 (0.93-1.78)	1.79 (1.16-2.75)	1.17 (1.02-1.33)	.02
Acute urinary retention					
Incidence rate, events/100 person-years (95% CI)	0.2 (0.1-0.4)	0.3 (0.2-0.5)	0.4 (0.2-0.8)	NA	NA
Unadjusted HR (95% CI)^b^	1 [Reference]	1.97 (0.81-4.78)	2.35 (0.81-6.81)	1.16 (0.87-1.55)	.33
Partially adjusted HR (95% CI)^c^	1 [Reference]	1.87 (0.75-4.62)	2.05 (0.68-6.21)	1.13 (0.84-1.54)	.42
Fully adjusted HR (95% CI)^d^	1 [Reference]	1.80 (0.70-4.62)	1.90 (0.56-6.39)	1.10 (0.77-1.56)	.60
Serious adverse events^e^					
Incidence rate, events/100 person-years (95% CI)	4.0 (3.3-4.7)	6.8 (6.3-7.4)	10.1 (8.8-11.6)	NA	NA
Unadjusted HR (95% CI)^b^	1 [Reference]	2.00 (1.65-2.42)	3.39 (2.71-4.24)	1.42 (1.34-1.50)	<.001
Partially adjusted HR (95% CI)^c^	1 [Reference]	1.93 (1.59-2.34)	3.15 (2.50-3.95)	1.39 (1.31-1.47)	<.001
Fully adjusted HR (95% CI)^d^	1 [Reference]	1.81 (1.48-2.23)	2.86 (2.21-3.69)	1.37 (1.27-1.47)	<.001

Abbreviations: BPH, benign prostatic hyperplasia; FI, frailty index; HR, hazard ratio; LUTS, lower urinary tract symptoms; NA, not applicable.

^a^ Clinical BPH progression was defined according to the original trial as the occurrence of any of the following: LUTS progression (an increase from baseline of at least 4 points in the American Urological Association Symptom Index), acute urinary retention (the inability to urinate requiring catheterization in the absence of an obvious cause of acute retention other than BPH, such as anesthesia), urinary tract infection or urosepsis, incontinence, or an increase in the serum creatinine level, attributable to benign prostatic hyperplasia, of at least 1.5 mg/dL (to convert to micromoles per liter, multiply by 88.4) and to a value at least 50% above baseline values.

^b^ HR and 95% CI were calculated using proportional hazards model. Linear *P* value was calculated using FI as continuous variable.

^c^ Adjusted for age, treatment group, prostate volume, postvoid residual, and maximum urinary flow rate.

^d^ Further adjusted for race and ethnicity, marital status, education, body mass index, and history of heart disease, hypertension, diabetes, pulmonary disease, neurological disease, and gastrointestinal disease. For the acute urinary retention model, no men with diabetes had an event so that covariate was removed and both race and ethnicity and education covariates were collapsed because of small cell sizes.

^e^ Serious adverse events were defined according to the original trial as fatal or life-threatening, permanently disabling, requiring or prolonging inpatient hospitalization, a congenital anomaly or cancer, an overdose, or medical events that jeopardize the patient and may require medical or surgical intervention to prevent a serious adverse event.

**Figure 2.**
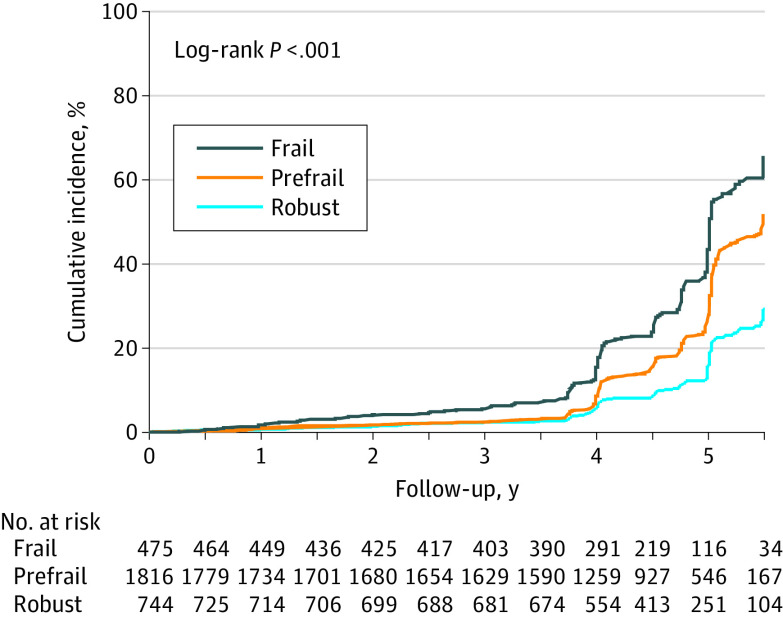
Cumulative Incidence Curve for Serious Adverse Events, by Frailty Status. Serious adverse events were defined according to the original trial as fatal or life-threatening, permanently disabling, requiring or prolonging inpatient hospitalization, a congenital anomaly or cancer, an overdose, or medical events that jeopardize the patient and may require medical or surgical intervention to prevent a serious adverse event.

Figure 2 displays the cumulative incidence curve for SAE, stratified by frailty status, and Table 2 reports the association of frailty with SAE (857 events). The incidence rate of SAEs was 4.0 events per 100 person-years among robust men, 6.8 events per 100 person-years among prefrail men, and 10.1 events per 100 person-years among frail men. Compared with robust men, the risk of SAE was almost 2 and 3 times higher among prefrail and frail men, respectively (prefrail vs robust AHR, 1.81; 95% CI, 1.48-2.23; frail vs robust AHR, 2.86; 95% CI, 2.21-3.69; linear *P* < .001). In sensitivity analyses restricted to men with 0 missing frailty index items, higher frailty index scores remained associated with greater risk of SAE (eTable 3 in the Supplement). There was no modification of the association between frailty and SAE by treatment group (*P* for interaction = .76) (eTable 4 in the Supplement).

Table 3 reports the association of frailty with clinical BPH progression stratified by treatment group. Although the test for interaction between frailty index and treatment group did not reach the a priori threshold of statistical significance (*P* for interaction between frailty index and treatment group = .06), the association of frailty with clinical BPH progression appeared to vary according to treatment group. Frailty was not associated with risk of clinical BPH progression among men randomized to placebo or finasteride monotherapy. However, among men randomized to doxazosin monotherapy, the AHR for clinical BPH progression was 2.64 (95% CI, 1.16-6.00) for frail vs robust men. In the combination therapy group, the AHR for clinical BPH progression was 2.46 (95% CI, 0.86-7.01) for frail vs robust men. The eFigure in the Supplement displays the cumulative incidence curve for clinical BPH progression stratified by frailty status and treatment group.

**Table 3.** Association of Frailty With Clinical Benign Prostatic Hyperplasia Progression, Stratified by Treatment Group^a^

Treatment group	Frailty status			Per 1 SD of FI	Linear *P* value
	Robust (FI score ≤0.1)	Prefrail (FI score 0.1-0.25)	Frail (FI score ≥0.25)		
Placebo					
Incidence rate, events/100 person-years (95% CI)	3.7 (2.6-5.3)	4.8 (3.8-5.9)	5.3 (3.5-8.2)	NA	NA
Fully adjusted HR (95% CI)^b^	1 [Reference]	1.40 (0.88-2.23)	1.57 (0.80-3.08)	1.05 (0.85-1.30)	.65
Finasteride					
Incidence rate, events/100 person-years (95% CI)	2.5 (1.6-3.9)	2.8 (2.2-3.7)	4.0 (2.6-6.3)	NA	NA
Fully adjusted HR (95% CI)^b^	1 [Reference]	1.12 (0.64-1.97)	1.46 (0.70-3.05)	1.20 (0.95-1.51)	.13
Doxazosin					
Incidence rate, events/100 person-years (95% CI)	1.7 (1.0-2.9)	2.9 (2.2-3.7)	4.2 (2.7-6.6)	NA	NA
Fully adjusted HR (95% CI)^b^	1 [Reference]	1.87 (0.98-3.57)	2.64 (1.16-6.00)	1.24 (0.98-1.58)	.08
Combination therapy (finasteride plus doxazosin)					
Incidence rate, events/100 person-years (95% CI)	0.8 (0.4-1.8)	1.4 (1.0-2.1)	2.8 (1.6-4.9)	NA	NA
Fully adjusted HR (95% CI)^b^	1 [Reference]	1.40 (0.59-3.35)	2.46 (0.86-7.01)	1.43 (1.07-1.91)	.02

Abbreviations: FI, frailty index; HR, hazard ratio.

^a^ Clinical benign prostatic hyperplasia progression was defined according to the original trial as the occurrence of any of the following: lower urinary tract symptoms progression (an increase from baseline of at least 4 points in the American Urological Association Symptom Index), acute urinary retention (the inability to urinate requiring catheterization in the absence of an obvious cause of acute retention other than benign prostatic hyperplasia, such as anesthesia), urinary tract infection or urosepsis, incontinence, or an increase in the serum creatinine level, attributable to benign prostatic hyperplasia, of at least 1.5 mg/dL (to convert to micromoles per liter, multiply by 88.4) and to a value at least 50% above baseline values.

^b^ HR and 95% CI were calculated using proportional hazards model adjusted for age, treatment group, prostate volume, postvoid residual, maximum urinary flow rate, race and ethnicity (collapsed to White vs all other races), marital status, education, body mass index, and history of heart disease, hypertension, diabetes, pulmonary disease, neurological disease, and gastrointestinal disease. Linear *P* value was calculated using FI as the continuous variable.

## Discussion

In this cohort study using data from a placebo-controlled randomized clinical trial, frail and prefrail men with LUTS due to suspected BPH were more likely to develop clinical BPH progression, primarily manifesting as increased LUTS severity, compared with robust men. This association was highest in the combination therapy (doxazosin plus finasteride) and doxazosin monotherapy groups. Frailty was similarly associated with greater risk of SAE, whether receiving placebo or drug therapy for BPH. These results suggest that among men with symptoms and objective measures suggestive of obstruction due to BPH, frailty is independently associated with worsening LUTS despite combination therapy. Frailty may, therefore, represent a novel mechanism of LUTS progression and medical BPH therapy failure. Although frail men receiving any drug had a higher risk of SAE compared with robust men, this risk was not significantly higher than among frail men randomized to placebo.

A growing body of evidence suggests that frail older men are more likely to report severe LUTS, overall as well as specific subtypes, compared with robust older men. Our team previously evaluated the association between phenotypic frailty (the presence of at least 3 of the components of weakness, slowness, weight loss, fatigue, and low physical activity^15^) and LUTS severity among 5979 community-dwelling older men enrolled in the Osteoporotic Fractures in Men Study.^33^ In that study, we found that the prevalence of moderate and severe LUTS was 46% and 13%, respectively, among frail men compared with 37% and 5% among robust men (moderate LUTS adjusted odds ratio, 1.4; 95% CI, 1.1-1.7; severe LUTS adjusted odds ratio, 2.5; 95%, 1.8-3.6).^33^ These associations were independent of age, comorbidities, multimorbidity, or LUTS treatments and persisted among men without urinary incontinence. A similar association was observed in a smaller study^34^ of older Korean men, where the prevalence of phenotypic frailty was 43% among men with severe LUTS, 16% among men with moderate LUTS, and 7% among men with no or mild LUTS. Phenotypic frailty and a higher deficit accumulation frailty index were both cross-sectionally associated with LUTS severity among older men and women in Japan.^23^ Positive associations have also been reported between measures of frailty and LUTS subtypes, such as overactive bladder,^35^ as well as physiological abnormalities that can cause LUTS, such detrusor overactivity^36^ and nocturnal polyuria.^24^ We previously demonstrated that compared with older men seeking treatment for other urologic conditions, older men with overactive bladder, mixed overactive bladder and BPH, and BPH were 2.6, 1.8, and 1.7 times, respectively, more likely to have slow Timed-Up-And-Go-Test times, an easily measurable surrogate for frailty.^21^ Our longitudinal analysis builds on this prior literature by reporting a positive association of deficit accumulation frailty index with a higher risk of clinical BPH progression and LUTS progression among men rigorously assessed for inclusion in a BPH trial and monitored carefully for progression events.

On the basis of current clinical guidelines, patients for whom maximal medical BPH therapy with combination therapy fails should be considered for more aggressive surgical interventions targeting the same mechanism of prostatic obstruction.^37^ However, our findings suggest that this algorithmic approach with sequential escalation of BPH interventions may frequently fail because the true cause of LUTS is rarely known and current surrogates of benign prostatic obstruction are neither sensitive nor specific. Accordingly, we believe that men who experience LUTS progression despite maximal medical BPH therapy could be experiencing progression due to mechanisms related to advanced biological age, manifesting as frailty, rather than prostatic obstruction. There is a critical need for diagnostic tests capable of distinguishing LUTS due to BPH and bladder outlet obstruction vs frailty and other systemic causes. The role of frailty as an indicator of surgical outcomes for BPH should also be evaluated.

The weight of prior literature and our results suggest that frailty is a systemic marker of biological age that potentially mediates the well-established association between chronological age and LUTS or BPH. Importantly, frailty and other systemic markers of increased biological age are currently not targeted by any existing male LUTS interventions^4,38^ and, thus, may represent a promising novel therapeutic target, particularly in men for whom maximal medical BPH therapy has failed. Consistent with this hypothesis, we observed evidence of a possible interaction between frailty status and treatment where frailty was most associated with clinical BPH progression among men receiving either combination therapy or doxazosin monotherapy. Frailty interventions often include physical activity, nutrition, multicomponent interventions, or individually tailored geriatric care models.^39,40,41,42,43^ Other interventions are designed to improve specific components of the frailty phenotype, such as resistance training to increase or maintain muscle mass among older adults,^44,45^ or to target frailty by interfering with upstream mechanisms of biological aging.^46,47,48^ Although targeting frailty may represent a novel approach to preventing or treating LUTS in older men, most frailty intervention studies have not measured LUTS severity, so it remains unknown whether preventing or reversing frailty will prevent LUTS progression.

To our knowledge, our analysis is the first to report a positive association between frailty based on deficit accumulation and SAE among men with LUTS due to suspected BPH. This is consistent with a large body of literature demonstrating substantial associations between frailty indices and morbidity, mortality, and adverse drug events.^28,30,49,50^ Contrary to our initial hypothesis, treatment group did not appear to modify the association between frailty and SAE (or vice versa). Therefore, the greater risk of SAE among frail men likely reflects the greater risk of morbidity and mortality among frail men in general, rather than the effect of any BPH drugs. Frail older men considering long-term medical BPH therapy should be counseled on their higher risk of SAE, albeit not specifically due to doxazosin or finasteride, and LUTS progression despite combination therapy. Current behavioral or surgical interventions with shorter time to benefit may be preferable for those who prefer to prioritize current quality of life and minimize polypharmacy.

### Strengths and Limitations

Strengths of this analysis included large sample size, adjudicated cases of clinical BPH progression, and SAE in the setting of a rigorously conducted clinical trial, repeated measurements using a reference standard LUTS questionnaire, and randomly assigned medical BPH therapies and placebo. This analysis also has important limitations. First, men were not randomized to their frailty status; therefore, unmeasured confounding is possible. However, we suspect that confounding by unmeasured factors, such as nutritional status or socioeconomic status, would be explained by demographic or clinical variables that we adjusted for in our multivariable model. Second, the MTOPS trial was conducted more than 2 decades ago, and doxazosin is an older generation α-blocker less commonly used than newer generation formulations, such as tamsulosin and alfuzosin.^51^ However, evidence from pooled analyses of randomized clinical trials suggests that older and newer generation α-blockers have similar efficacy, whereas newer generation α-blockers generally have fewer adverse effects.^52^ Clinical guidelines for LUTS due to suspected BPH have also remained unchanged since the MTOPS trial was published; combination therapy with any generation of α-blocker plus a 5α-reductase inhibitor is still first-line treatment for preventing clinical BPH progression.^3,53^ We do not have reason to expect that the association between frailty and clinical BPH progression or SAE has changed since the data were collected, although it is possible that the reporting of LUTS and a small subset of subjective frailty index items has changed over time as a result of evolving socioecological norms. Third, men enrolled in MTOPS were predominantly younger than 70 years, healthy enough to enroll in a BPH trial, and White; therefore, generalizability is unknown for an older, more ill, or more racially diverse population of men with LUTS due to suspected BPH. Fourth, although we believed that it was important to formally test for interactions between frailty and treatment group, our power was limited to detect statistically significant yet clinically meaningful interactions. Fifth, we were only able to assess deficit accumulation frailty.

## Conclusions

In conclusion, a discussion of both potential benefits and harms via shared decision-making is needed before initiating medical BPH therapy in this population of older frail men. In particular, such patients should be counseled regarding their higher risk of LUTS progression despite combination therapy and their likelihood of experiencing SAE regardless of treatment choice. Frailty-related mechanisms of LUTS progression should be investigated, and LUTS should be assessed in future frailty intervention trials. Ultimately, interventions targeting frailty may represent a novel approach to preventing LUTS progression in older men.

## Notes

Supplement. **eTable 1.** Items and Scoring Scheme for the MTOPS Frailty Index **eTable 2.** Mean Change in AUA Symptom Index Among Men With Lower Urinary Tract Symptom Progression, Overall and by Frailty Status **eTable 3.** Association of Frailty With Clinical Benign Prostatic Hyperplasia (BPH) Progression, Lower Urinary Tract Symptoms (LUTS) Progression, Acute Urinary Retention, and Serious Adverse Events Among Men With 0 Missing Frailty Index Items **eTable 4.** Cumulative Incidence Rate of Serious Adverse Events, by Treatment Arm and Frailty Status **eFigure.** Cumulative Incidence Curves for Clinical Benign Prostatic Hyperplasia (BPH) Progression, by Frailty Status and Treatment Arm
