# Supplementary material for: Assessment of Frailty and Association With Progression of Benign Prostatic Hyperplasia Symptoms and Serious Adverse Events Among Men Using Drug Therapy
Source: JAMA Netw Open. 2021 Nov 24;4(11):e2134427. doi: 10.1001/jamanetworkopen.2021.34427 (PMC8613596; doi:10.1001/jamanetworkopen.2021.34427)
Supplement: Supplement. — eTable 1. Items and Scoring Scheme for the MTOPS Frailty Index eTable 2. Mean Change in AUA Symptom Index Among Men With Lower Urinary Tract Symptom Progression, Overall and by Frailty Status eTable 3. Association of Frailty With Clinical Benign Prostatic Hyperplasia (BPH) Progression, Lower Urinary Tract Symptoms (LUTS) Progression, Acute Urinary Retention, and Serious Adverse Events Among Men With 0 Missing Frailty Index Items eTable 4. Cumulative Incidence Rate of Serious Adverse Events, by Treatment Arm and Frailty Status eFigure. Cumulative Incidence Curves for Clinical Benign Prostatic Hyperplasia (BPH) Progression, by Frailty Status and Treatment Arm [file jamanetwopen-e2134427-s001.pdf]

Supplemental Online Content

Bauer SR, Walter LC, Ensrud KE, et al. Assessment of frailty and association with progression of benign prostatic hyperplasia symptoms and serious adverse events among men using drug therapy. *JAMA Netw Open*. 2021;4(11):e2134427. doi:10.1001/jamanetworkopen.2021.34427

eTable 1. Items and Scoring Scheme for the MTOPS Frailty Index
eTable 2. Mean Change in AUA Symptom Index Among Men With Lower Urinary Tract Symptom Progression, Overall and by Frailty Status
eTable 3. Association of Frailty With Clinical Benign Prostatic Hyperplasia (BPH) Progression, Lower Urinary Tract Symptoms (LUTS) Progression, Acute Urinary Retention, and Serious Adverse Events Among Men With 0 Missing Frailty Index Items
eTable 4. Cumulative Incidence Rate of Serious Adverse Events, by Treatment Arm and Frailty Status
eFigure. Cumulative Incidence Curves for Clinical Benign Prostatic Hyperplasia (BPH) Progression, by Frailty Status and Treatment Arm

This supplemental material has been provided by the authors to give readers additional information about their work.

© 2021 Bauer SR et al. *JAMA Network Open.*

eTable 1. Items and Scoring Scheme for the MTOPS Frailty Index.

Item #	Source	Item	Response	Score	Notes
1	SPRINT	Self-rated general health	Excellent	0	
			Very Good	0.25	
			Good	0.5	
			Fair	0.75	
			Poor	1	
2	MTOPS	Self-rated general health (compared to one year ago)	Excellent	0	
			Very Good	0.25	
			Good	0.5	
			Fair	0.75	
			Poor	1	
3	MTOPS	Does your health limit you now in vigorous activities, such as running, lifting heavy objects, participating in strenuous sports?	No, not limited at all	0	
			Yes, limited a little	0.5	
			Yes, limited a lot	1	
4	SPRINT	Does your health limit you now in moderate activities, such as moving a table, pushing a vacuum cleaner, bowling, or playing golf?	No, not limited at all	0	
			Yes, limited a little	0.5	
			Yes, limited a lot	1	
5	SPRINT	Does your health limit you now in climbing several flights of stairs?	No, not limited at all	0	
			Yes, limited a little	0.5	
			Yes, limited a lot	1	
6	MTOPS	Does your health limit you now in climbing one flight of stairs?	No, not limited at all	0	
			Yes, limited a little	0.5	
			Yes, limited a lot	1	
7	SPRINT	During the past 4 weeks, how much did pain interfere with your normal work?	Not at all	0	
			A little bit	0.25	
			Moderately	0.5	
			Quite a bit	0.75	
			Extremely	1	
8	SPRINT	How much of the time during the past 4 weeks did you have a lot of energy?	All of the time	0	
			Most of the time	0.2	
			A good bit of the time	0.4	
			Some of the time	0.6	
			A little of the time	0.8	
			None of the time	1	

© 2021 Bauer SR et al. *JAMA Network Open.*

Item #	Source	Item	Response	Score	Notes
9	SPRINT	How much of the time during the past 4 weeks have you felt downhearted and blue?	None of the time<br>A little of the time<br>Some of the time<br>A good bit of the time<br>Most of the time<br>All of the time	0<br>0.2<br>0.4<br>0.6<br>0.8<br>1	
10	SPRINT	During the past 4 weeks, how much of the time has your physical health or emotional problems interfered with your social activities?	None of the time<br>A little of the time<br>Some of the time<br>Most of the time<br>All of the time	0<br>0.25<br>0.5<br>0.75<br>1	
11	MTOPS	How much of the time during the past 4 weeks have you felt full of pep?	All of the time<br>Most of the time<br>Some of the time<br>A little of the time<br>None of the time	0<br>0.25<br>0.5<br>0.75<br>1	
12	MTOPS	How much of the time during the past 4 weeks have you felt so down in the dumps that nothing could cheer you up?	None of the time<br>A little of the time<br>Some of the time<br>Most of the time<br>All of the time	0<br>0.25<br>0.5<br>0.75<br>1	
13	MTOPS	How much of the time during the past 4 weeks have you felt worn out?	None of the time<br>A little of the time<br>Some of the time<br>Most of the time<br>All of the time	0<br>0.25<br>0.5<br>0.75<br>1	
14	MTOPS	How much of the time during the past 4 weeks have you felt tired?	None of the time<br>A little of the time<br>Some of the time<br>Most of the time<br>All of the time	0<br>0.25<br>0.5<br>0.75<br>1	
15	MTOPS	Does your health limit you now in lifting or carrying groceries?	No, not limited at all<br>Yes, limited a little<br>Yes, limited a lot	0<br>0.5<br>1	
16	MTOPS	Does your health limit you now in bending, kneeling, or stooping?	No, not limited at all<br>Yes, limited a little<br>Yes, limited a lot	0<br>0.5<br>1	
17	MTOPS	Does your health limit you now in walking more than a mile?	No, not limited at all<br>Yes, limited a little<br>Yes, limited a lot	0<br>0.5<br>1	

© 2021 Bauer SR et al. *JAMA Network Open.*

Item #	Source	Item	Response	Score	Notes
18	MTOPS	Does your health limit you now in several blocks?	No, not limited at all<br>Yes, limited a little<br>Yes, limited a lot	0<br>0.5<br>1	
19	MTOPS	Does your health limit you now in walking one block?	No, not limited at all<br>Yes, limited a little<br>Yes, limited a lot	0<br>0.5<br>1	
20	MTOPS	Does your health limit you now in bathing or dressing yourself?	No, not limited at all<br>Yes, limited a little<br>Yes, limited a lot	0<br>0.5<br>1	
21	MTOPS	During the past 4 weeks, have you had any of the following problems with your work or other regular daily activities as a result of your physical health?	Cut down the amount of time you spent on work or other activities	1	
22	SPRINT		Accomplished less than you would like	1	SPRINT: 5-level response variable
23	MTOPS		Were limited in the kind of work or other activities	1	
24	MTOPS		Had difficulty performing the work or other activities	1	
25	MTOPS	During the past 4 weeks, have you had any of the following problems with your work or other regular daily activities as a result of any emotional problems (such as feeling depressed or anxious)?	Cut down the amount of time you spent on work or other activities	1	
26	MTOPS		Accomplished less than you would like	1	
27	SPRINT		Didn't do work or other activities as carefully as usual	1	SPRINT: 5-level response variable
28	MTOPS	How much bodily pain have you had during the past 4 weeks?	None<br>Very mild<br>Mild<br>Moderate<br>Severe<br>Very severe	0<br>0.2<br>0.4<br>0.6<br>0.8<br>1	
29	SPRINT	Self-reported history of diabetes		1	
30	SPRINT	Self-reported history of heart disease	Yes	1	SPRINT: heart attack, heart failure, atrial fibrillation, and angina assessed separately

© 2021 Bauer SR et al. *JAMA Network Open.*

31	SPRINT	Self-reported history of cancer	Yes	1	

Item #	Source	Item	Response	Score	Notes
32	MTOPS	Self-reported history of lung disease	Yes	1	
33	MTOPS	Self-reported history of rheumatologic/vascular disease	Yes	1	
34	MTOPS	Self-reported history of liver disease	Yes	1	
35	MTOPS	Self-reported history of endocrine disease	Yes	1	
36	MTOPS	Self-reported history of gastrointestinal disease	Yes	1	
37	MTOPS	Self-reported history of neurologic disease	Yes	1	
38	MTOPS	Self-reported history of hematologic disease	Yes	1	
39	MTOPS	Self-reported history of anemia	Yes	1	
40	MTOPS	Self-reported history of hypertension	Yes	1	
41	MTOPS	Self-reported history of skin disease	Yes	1	
42	MTOPS	Self-reported history of gross hematuria	Yes	1	
43	MTOPS	Self-reported history of microscopic hematuria	Yes	1	
44	MTOPS	Self-reported history of renal disease	Yes	1	
45	SPRINT	Chronic Kidney Disease	eGFR <60 ml/min/1.73m^2	1	Based on CKD-EPI Creatine Equation (2009)[9]
46	MTOPS	Proteinuria	Absent<br>Present	0<br>1	Assessed via dipstick
47	SPRINT	Total Cholesterol	<3.5 or >7 mmol/l	1	
48	SPRINT	Potassium	<3.5 or >6 mmol/l	1	
49	SPRINT	Sodium	<135 or 150 mmol/l	1	
50	SPRINT	Glucose	<2.8 or >11 mmol/l	1	
51	SPRINT	Blood Urea Nitrogen	<3 or >7.5 mmol/l	1	
52	LAB FI	Albumin	<3.2 or >4.5 g/dL	1	
53	LAB FI	Hemoglobin	<13.5 or >18 g/dL	1	
54	LAB FI	Platelet count	<150 or >450 1000cells/uL	1	
55	LAB FI	Bicarbonate	<21 or >28 mmol/L	1	
56	LAB FI	Uric acid	<240 or >510 umol/L	1	

© 2021 Bauer SR et al. *JAMA Network Open.*

Item #	Source	Item	Response	Score	Notes
57	LAB FI	Calcium	<2.3 or >2.74 mmol/L	1	
58	LAB FI	Phosphorus	<0.74 or >1.52 mmol/L	1	
59	LAB FI	Alkaline Phosphatase	<20 or >130 U/L	1	
60	LAB FI	Total Bilirubin	<2 or >21 umol/L	1	
61	LAB FI	Total Protein	<60 or >78 g/L	1	
62	LAB FI	Pulse Pressure	<30 or >65 mmHg	1	
63	LAB FI	Polypharmacy	0-4 medications	0	
			5-9 medications	0.5	
			≥10 medications	1	
64	SPRINT	Overweight / Obesity	BMI ≥ 25 but <30 kg/m^2	0.5	
			BMI ≥ 30 kg/m2	1	
65	SPRINT	Underweight	BMI < 18.5 kg/m^2	1	
66	LAB FI	Systolic Blood Pressure	<140 mmHg	0	SPRINT: 1 point for ≥ 180 mmHg
			140-159 mmHg	0.33	
			160-179 mmHg	0.67	
			≥ 180 mmHg	1	
67	SPRINT	Diastolic Blood Pressure	≥ 90 mmHg	1	
68	LAB FI	Heart Rate	<60 or >99 beats/min	1	

SPRINT: Items included based on frailty index from the Systolic Blood Pressure Intervention Trial.
LAB FI: Items included based on frailty index using common laboratory values from Blodgett et al.
MTOPS: Items unique to MTOPS not included in the SPRINT for LAB FI frailty indices.

eTable 2. Mean Change in AUA Symptom Index Among Men With Lower Urinary Tract Symptom Progression, Overall and by Frailty Status.

	**Robust (FI S0.1)**	**Pre-frail (FI 0.1-0.25)**	**Frail (FI ≥0.25)**	**Overall**
Number of LUTS Progression Events	56	161	57	
Change in AUA Symptom Index from Baseline to Progression Event, mean ±SD				
Total Score	7.4 ±3.3	6.7 ±2.9	7.0 ±2.8	6.9 ±2.9
Voiding Sub-score	4.9 ±2.8	4.0 ±2.4	4.1 ±2.5	4.2 ±4.2
Storage Sub-score	2.5 ±2.3	2.8 ±2.0	2.9 ±1.9	2.8 ±2.1

FI frailty index; LUTS lower urinary tract symptoms; AUA American Urological Association; SD standard deviation

© 2021 Bauer SR et al. *JAMA Network Open.*

eTable 3. Association of Frailty With Clinical Benign Prostatic Hyperplasia (BPH) Progression, Lower Urinary Tract Symptoms (LUTS) Progression, Acute Urinary Retention, and Serious Adverse Events Among Men With 0 Missing Frailty Index Items.

	Frailty Status				
	**Robust (FI S0.1)**	**Pre-frail (FI 0.1-0.25)**	**Frail (FI ≥0.25)**	**Per 1 SD of FI**	**Linear *P* value**
**Clinical BPH Progression***					
Unadjusted HR[††] (95% CI)	1.00 (Ref.)	1.20 (0.90, 1.60)	1.55 (1.08, 2.24)	1.15 (1.03, 1.29)	0.01
Fully Adjusted[¶] HR (95% CI)	1.00 (Ref.)	1.26 (0.92, 1.72)	1.61 (1.05, 2.46)	1.19 (1.04, 1.36)	0.01
**LUTS Progression**					
Unadjusted HR[††] (95% CI)	1.00 (Ref.)	1.11 (0.81, 1.52)	1.42 (0.95, 2.14)	1.14 (1.01, 1.29)	0.04
Fully Adjusted[¶] HR (95% CI)	1.00 (Ref.)	1.19 (0.84, 1.67)	1.51 (0.94, 2.43)	1.18 (1.01, 1.37)	0.03
**Acute Urinary Retention**					
Unadjusted HR[††] (95% CI)	1.00 (Ref.)	1.62 (0.61, 4.29)	1.77 (0.54, 5.81)	1.12 (0.81, 1.57)	0.48
Fully Adjusted[¶] HR (95% CI)	1.00 (Ref.)	1.34 (0.48, 3.77)	1.29 (0.33, 5.04)	1.06 (0.97, 1.08)	0.41
**Serious Adverse Events**[†]					
Unadjusted HR[††] (95% CI)	1.00 (Ref.)	1.77 (1.44, 2.18)	2.69 (2.12, 3.42)	1.32 (1.25, 1.41)	<0.001
Fully Adjusted[¶] HR (95% CI)	1.00 (Ref.)	1.55 (1.24, 1.93)	2.10 (1.60, 2.76)	1.25 (1.16, 1.35)	<0.001

FI frailty index; HR hazard ratio; SD standard deviation

* Clinical BPH progression defined according to the original trial as the occurrence of any of the following: LUTS progression (an increase from base line of at least 4 points in the American Urological Association Symptom Index), acute urinary retention (the inability to urinate requiring catheterization in the absence of an obvious cause of acute retention other than BPH, such as anesthesia), urinary tract infection or urosepsis, incontinence, or an increase in the serum creatinine level, attributable to benign prostatic hyperplasia, of at least 1.5 mg per deciliter and to a value at least 50 percent above base-line values.

† Serious adverse event defined according to the original trial as fatal or life threatening, permanently disabling, requiring or prolonging inpatient hospitalization, a congenital anomaly or cancer, an overdose, or medical events that jeopardize the patient and may require medical or surgical intervention to prevent a serious adverse event.

†† HR and 95% CI calculated using proportional hazards model. Linear *P* value calculated using frailty index as continuous variable.

¶ Adjusted for age, treatment arm, prostate volume, post-void residual, and maximum urinary flow rate, race/ethnicity, marital status, education, body mass index, and history of heart disease, hypertension, diabetes mellitus, pulmonary disease, neurologic disease, and gastrointestinal disease. For the acute urinary retention model, zero men with diabetes had an event so that covariate was removed and both race/ethnicity and education covariates were collapsed due to small cell sizes.

eTable 4. Cumulative Incidence Rate of Serious Adverse Events, by Treatment Arm and Frailty Status.

	Treatment Arm			
	**Placebo**	**Finasteride (F)**	**Doxazosin (D)**	**Combination Therapy (F+D)**
	Serious Adverse Event* Incidence Rate /100 person-years (95% CI)			
**Robust (FI S0.1)**	3.9 (2.8, 5.5)	4.2 (3.0, 5.9)	4.0 (2.8, 5.6)	3.8 (2.7, 5.3)
**Pre-frail (FI 0.1-0.25)**	6.5 (5.4, 7.8)	7.3 (6.2, 8.6)	7.3 (6.2, 8.6)	6.2 (5.2, 7.4)
**Frail (FI ≥0.25)**	9.9 (7.3, 13.2)	8.2 (6.1, 11.1)	9.7 (7.3, 12.9)	12.7 (9.9, 16.3)

FI frailty index

* Serious adverse event defined according to the original trial as fatal or life threatening, permanently disabling, requiring or prolonging inpatient hospitalization, a congenital anomaly or cancer, an overdose, or medical events that jeopardize the patient and may require medical or surgical intervention to prevent a serious adverse event.

© 2021 Bauer SR et al. *JAMA Network Open.*

eFigure. Cumulative Incidence Curves for Clinical Benign Prostatic Hyperplasia (BPH) Progression, by Frailty Status and Treatment Group.

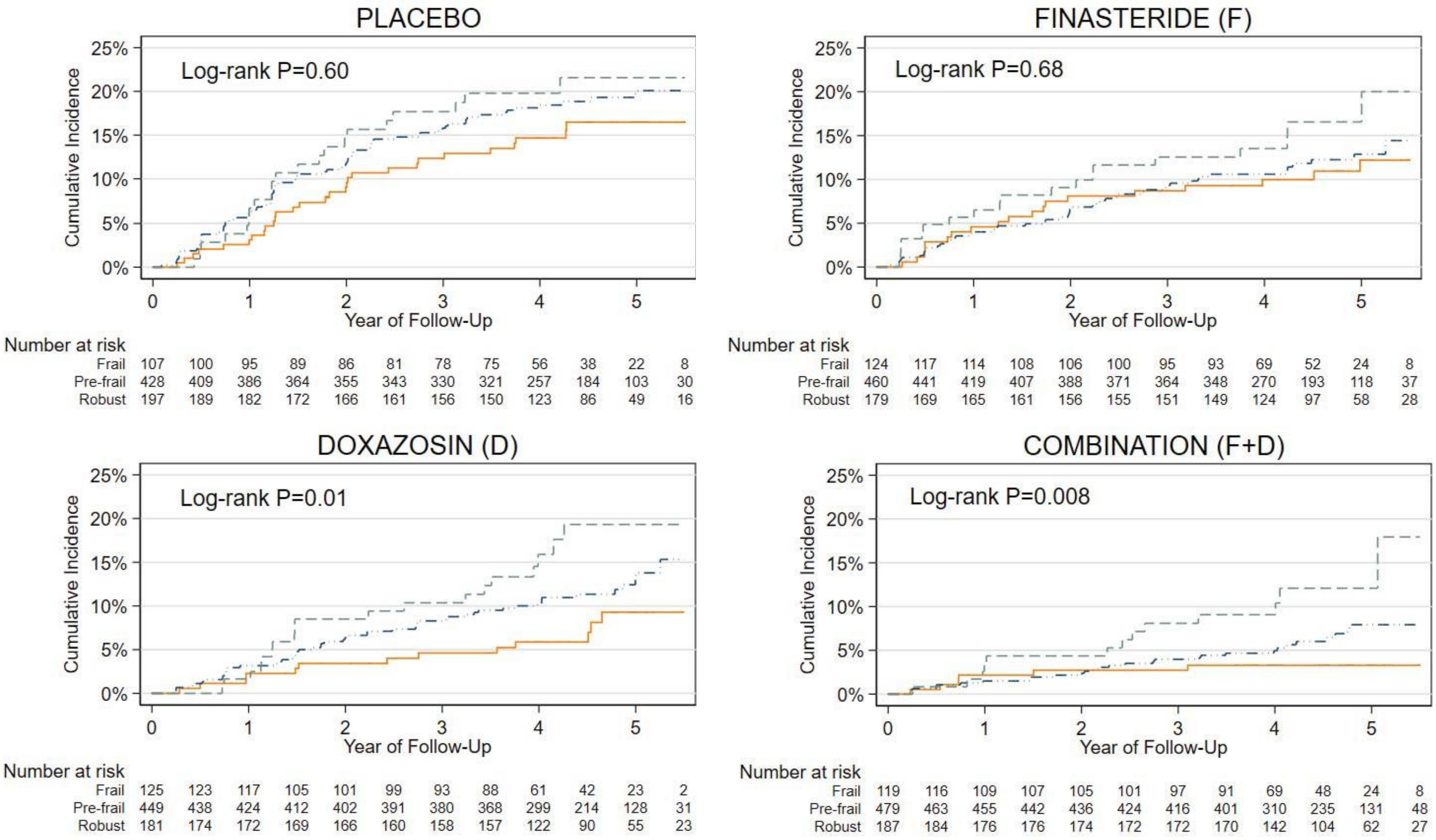

© 2021 Bauer SR et al. *JAMA Network Open.*
